# Advances in the Production and Batch Reformatting of Phage Antibody Libraries

**DOI:** 10.1007/s12033-019-00207-0

**Published:** 2019-08-29

**Authors:** Rose H. Reader, Robert G. Workman, Ben C. Maddison, Kevin C. Gough

**Affiliations:** 1 School of Veterinary Medicine and Science, The University of Nottingham, College Rd., Sutton Bonington, Loughborough, Leicestershire, LE12 5RD UK; 2 ADAS Biotechnology, School of Veterinary Medicine and Science, The University of Nottingham, College Rd., Sutton Bonington, Loughborough, Leicestershire, LE12 5RD UK

**Keywords:** Phage display, Recombinant antibody, scFv, Fab, Nanobody, V_H_H, V_NAR_

## Abstract

Phage display antibody libraries have proven an invaluable resource for the isolation of diagnostic and potentially therapeutic antibodies, the latter usually being antibody fragments converted into IgG formats. Recent advances in the production of highly diverse and functional antibody libraries are considered here, including for Fabs, scFvs and nanobodies. These advances include codon optimisation during generation of CDR diversity, improved display levels using novel signal sequences, molecular chaperones and isomerases and the use of highly stable scaffolds with relatively high expression levels. In addition, novel strategies for the batch reformatting of scFv and Fab phagemid libraries, derived from phage panning, into IgG formats are described. These strategies allow the screening of antibodies in the end-use format, facilitating more efficient selection of potential therapeutics.

## Introduction

Phage display libraries based on M13 filamentous bacteriophage can present a vast diversity of antibody fragments on the pIII minor coat protein, utilising either a phagemid or phage vector system to encode the antibody-coat protein fusion (the different display systems including mutated helper phage are reviewed elsewhere, [1–3]). The presence of the antibody on the surface of the phage particle is accompanied by the gene for the antibody being packaged within the phage. In this way the phenotype (antibody binding) and the genotype (antibody gene) are linked [2]. Within standard panning methods, the highly diverse antibody library is introduced to an immobilised antigen and unbound phage washed away, bound phage are then eluted, usually by a shift in pH and the resulting sub-library propagated in *Escherichia coli* to amplify each phage clone. Such panning cycles are usually carried out 3–5 times to enrich for phage-antibodies that bind to the target antigen and hundreds of single phage clones are then isolated, propagated and tested in ELISAs [2]. More recently, screening has used next generation sequencing platforms to sequence thousands to millions of antibody genes contained within the selected sub-libraries of phage after panning. Sequencing usually targets the whole VH or the CDRH3 domain. These methods allow a thorough investigation of the enrichment of phage clones, even when they are minor components of the sub-libraries [4–6]. Phage display libraries can enable the identification of a variety of antibodies with desirable binding properties including antibodies that bind to toxic or non-immunogenic antigens.

**Fig. 1:**
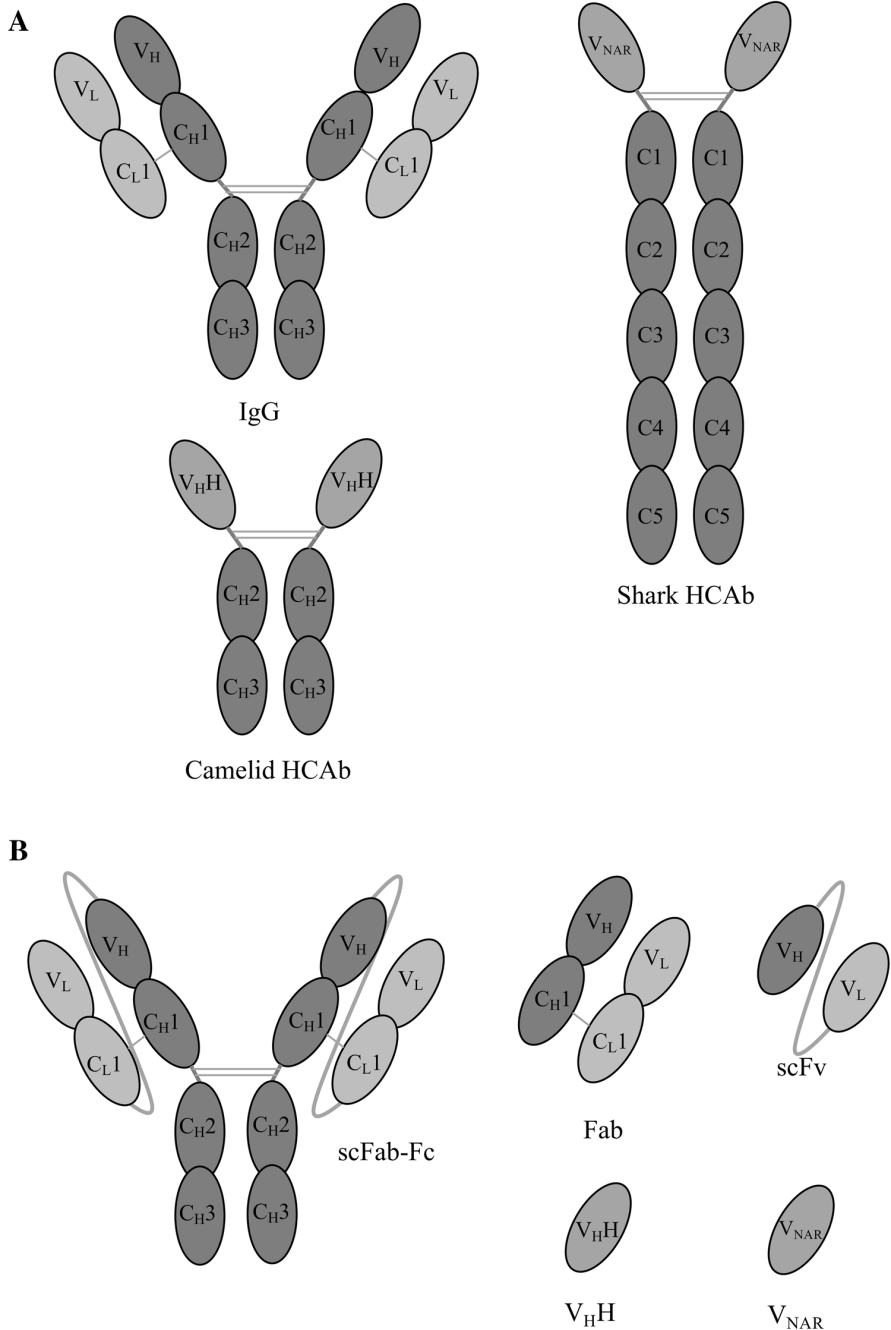
Representations of the different structures of antibodies and antibody fragments for phage display. The multi-domain structure of conventional IgG and heavy chain antibody (camelid and shark) (**a**). Antibody fragments can be displayed on phage as Fabs, scFvs or nanobodies (V_H_Hs or V_NAR_, derived from camelid or shark, respectively). The synthetic scFab-Fc dimer is also shown (**b**). *V* variable domain, *C* constant domain, *H* heavy chain, *L* light chain

Naturally occurring or designed antibody domains have been developed that are suited for phage display but still retain the binding properties of a full-length IgG (Fig. 1). These domains include antigen binding fragments (Fab), single chain Fv (scFv) and single domain antibodies (nanobodies). The latter include camelid V_H_H and shark V_NAR_ (New Antigen Receptor).

Phage antibody libraries are classed as naïve, semi-synthetic, synthetic or immune libraries [2]. The latter are produced by cloning the antibody genes from immunised animals (e.g. [7–9]) or diseased or vaccinated humans [10, 11] and are directed towards a single or limited number of antigens. Naïve phage display libraries are produced from the natural antibody repertoire of donors. Alternatively, phage display libraries can be produced using a relatively small diversity of natural antibody sequences whose diversity is increased through mutating one or more complementarity determining regions (CDRs) (semi-synthetic libraries). Libraries can also be based on a single or very low number of “scaffold” sequences with introduced diversity in the CDRs (synthetic libraries). Immune libraries require immunisation and library cloning for each antigen but small repertoire libraries (~ 10^6^) usually facilitate the isolation of high affinity binders (e.g. [7–9]). On the other hand, naïve or synthetic/semi-synthetic libraries (collectively known as single-pot libraries) can be used against any antigen but are required to have very high diversity (typically > 10^10^) to allow the isolation of high affinity binders (e.g. [12–16]).

In terms of antibody formats, Fabs consist of the VH-CH1 and VL-CL chains, one of which is fused to the pIII protein [17]. In phage display systems these two chains are usually expressed separately and assemble into the Fab format within the periplasm of the bacteria via a disulphide bond linkage. ScFvs consist of just the VH and VL chains joined together by a peptide linker with one chain fused to the pIII protein [12]. This single chain is expressed and targeted to the periplasm where the scFv folds into its active conformation. Nanobodies are an alternative antibody fragment and at just 12–15 kDa are smaller than Fabs or scFvs. They are derived from natural heavy chain antibodies found in camelids and sharks that lack a light chain (Fig. 1a). Nanobodies consist of the equivalent of a conventional IgG VH domain and again can be fused to the pIII phage protein for display [18, 19].

Examples of all of these antibody types with high specificity and affinities in the low nM to pM range have been directly selected by phage display (Table 1). A consideration when developing a single-pot antibody phage display system is that a high diversity will be more likely to deliver the best binders and the higher the level of display the more effective the selection of these binders will be. High specificity and affinities enable them to be effective in their applications such as for drug delivery, therapeutics, in vivo imaging or diagnostic assays. Recombinant antibody fragments are relatively small and lack an Fc domain. Considering in vivo applications, these properties may have advantages where high tumour/tissue penetration and a short half-life are advantageous, such as for in vivo imaging [20, 21]. In contrast, most protein therapeutics are based on human/humanised antibody fragments that are converted into larger antibody structures such as IgG. A strategy designed to minimise immunogenicity, elicit effector functions and extend serum half-life [22]. The use of recombinant antibodies as therapeutics is of increasing importance. In a review in 2016, Frenzel et al. reported that there were 64 phage display derived antibodies or antibody conjugates that had undergone some stage of clinical trials and the vast majority of these antibody fragments had been converted to IgG or IgG-conjugate formats [23]. A more recent update in 2019 stated that over 80 phage display derived antibodies had entered clinical studies and more than 10 had marketing approval [24].

**Table 1:** Comparing high affinity ligand types that can be used in phage display

Ligand type	~Size (kDa)	Typical affinity range of selected clones*	Reference examples
Antigen binding fragment (Fab)	55	nM-pM	[29, 100]
Single chain variable fragment (scFv)	25	nM-pM	[12, 35]
V_H_H	15	nM-pM	[101, 102]
Shark V_NAR_	12	nM	[19, 90]
Human VH or VL single domain antibodies	15	μM-nM	[64–66]

*Before affinity maturation

This review will consider recent advances in the cloning and functional display of antibody libraries, as well as their batch reformatting into IgG-like molecules.

## Antigen Binding Fragment (Fab)

Fabs were one of the first type of recombinant antibody that was produced as a diverse repertoire displayed on the surface of bacteriophage, first being described in 1991 [17]. As mentioned above, antibodies will usually be required to have high specificity and affinity and therapeutic applications may require the antibody to be converted into an IgG format. Ideally, this reformatting would be done before the screening of antibodies resulting from phage display panning as the binding properties of the monovalent Fab will be different from the divalent IgG [21, 25, 26]. In addition, high expression levels and high stability of Fab and IgG are vital to produce useful reagents [25].

There have been several recent reports on the production of Fab phage display libraries with either simplified cloning strategies or improved/distinct diversity. Typically, the light chain VL-CL gene will be expressed separately from the VH-CH1-pIII fusion gene. Both proteins are targeted to the periplasm where they interact to form a Fab-pIII fusion that is then packaged into assembling phage.

Type II restriction enzymes have been used in a so-called ‘seamless’ cloning strategy for Fabs. After screening human, rabbit and mouse antibody gene repertoires, enzymes were selected that either did not cut or cut very infrequently in antibody gene sequences. Appropriate phagemid vectors were then produced including these restriction enzyme cleavage sites for the cloning of human (using *Bsm*BI and *Sap*I, vector pUP-22Hb), rabbit (*Sap*I only, vector pUP-22Rc) and mouse (*Sap*I, vector pUP-22Mc) Fabs, and libraries of diversities ~ 10^9^ produced [27]. These libraries only use kappa light chains and this strategy clones amplicons of VH and VL into the vector in a single step. Whilst this was applied to produce immune libraries only (the human library was a mouse/human chimeric library), the repertoire achieved for the libraries demonstrates the strategy is applicable to produce more diverse single-pot Fab libraries.

It has been reported that rabbit B cell repertoires may be very different to those of human or mice due to the unique ontogeny of rabbit B cells and extensive somatic gene conversion [28]. Peng et al. [29], recently produced a naïve rabbit/human chimeric Fab library of ~ 10^10^ diversity made up of rabbit VH/VL regions and human CH1 and CL domains. The strategy used multiple step wise PCR amplifications of the VH and VL genes for use as part of the templates in a 3-fragment overlap extension PCR to amplify VL-CL-VH for Kappa and Lambda light chains, and the subsequent cloning of the products into a CH1 containing vector. The study selected a range of antibodies with high specificity and affinities in the low nM range.

When synthetic diversity is added into libraries it is usually targeted at the CDRH3 and CDRL3 domains as they are reported to have the greatest influence on specificity and affinity [30]. One recently produced library used the scaffold Fab Hu4D5-8 that has high thermal stability. Limited diversity was introduced into the CDR1 and CDR2 of both heavy and light chains with sequences matching the most canonical CDR conformations in the scaffold sequence. High random sequence diversity was then introduced into CDR3s (7–25 residues) to produce a library repertoire of 7 × 10^9^. The strategy also used a previously described bivalent display of Fabs which are displayed in a Fab-GCN4 leucine zipper-pIII display format and includes intermolecular disulphide bond sites [31]. Fabs were isolated with ~ 2 nM affinities.

There are several commercially available Fab libraries, for example the Ylanthia library from MorphoSys [30]. This library has very high diversity and is based on 36 fixed heavy/light chain pairs selected from 400 combinations that were characterised for desirable biophysical properties. These were relatively high expression in bacteria and mammalian cells as Fabs or IgG, respectively, high display levels on phage, high thermal stability and low aggregation in both Fab and IgG formats. CDRH1 and CDRH2 had limited diversity introduced to remove sites for post-translational modifications (PTM). High diversity was then introduced into CDRH3 and CDRL3, again avoiding PTM sites, to produce a library of 10^11^ clones. One clone selected by panning had an affinity of 700 pM. A range of clones gave yields ranging from 1.5–13 mg/L and when converted into IgG and transiently expressed in HKB11 cells, 20–80 mg/L IgG was produced.

Stability of the Fab and concomitant IgG is an important factor in library design and can be addressed as above by selecting scaffold Fab structures with high thermostability. An alternative approach isolated thermostable scaffolds by transiently heating a Fab phage library before selection of functional Fabs with an anti-light chain antibody [32]. Five unique Fabs were identified and each had increased stability. Combinations of the Fabs’ mutations resulted in an optimal triple mutant with high stability that also translated into a similarly highly stable IgG. It was speculated that the CDRH1 region mediated this increase in stability.

Insufficient solubility can often lead to candidate antibodies not being fully developed for diagnostic or therapeutic applications. In silico programmes that can predict antibody solubility have been applied to Fabs that have been converted into IgGs. The predictions from these in silico programmes have been compared to a range of in vitro tests for protein aggregation, stability and solubility [33]. Seventeen antibody variants with a wide range of predicted solubilities were characterised and the in silico prediction tools displayed significant correlation with the in vitro test. The authors concluded that the CamSol programme could be used to efficiently identify lead antibodies to take forward for development. This provides a rapid and cost-effective method for improved selection of lead antibodies. Such in silico stability prediction tools may also have application to the design of a high solubility Fab scaffold for synthetic library production. Whilst the selection of such a scaffold may help facilitate the selection of high solubility reagents, this may be limited by the mutated CDR regions making significant contributions to the solubility of the Fabs. Post-panning solubility testing will therefore still be required.

Fabs are often reported as having relatively low phage display levels compared to scFvs, which may be attributed to their relatively complex disulphide bond formation [26, 34]. Several recent studies have looked to improve Fab display levels. Huovinen et al. [35] compared the panning enrichment of the same Fab repertoire on pIII (rescued with wild type helper phage or hyperphage), truncated pIII and pIX (a minor coat with ~ 5 copies per phage particle, the same as for pIII). They also compared scFv displayed on truncated pIII. Fab displayed on pIX had the lowest selection efficiency, the highest diversity of clones was obtained with Fab or scFvs displayed on truncated pIII. Increasing the display valency with hyperphage improved the selected clone diversity. It was shown that a single propagation of phage resulted in large reductions in diversity of antibodies for the Fab-pIII and scFv-truncated pIII libraries, where frameshift mutations seemed to be selected for. The study indicated that the optimal display of Fabs was on truncated pIII and this may be further improved by increasing Fab valency using hyperphage.

The use of co-expression of molecular chaperones or isomerases to improve Fab folding/stability and export into the periplasm has also recently been reported. For example, co-plasmid expression of *DsbA*, *DsbC*, *FkpA* and *SurA* has been used. The expression of the peptidyl prolyl cis–trans isomerases (PPIases) *FkpA* and *SurA* directed to the periplasm may improve folding, and *DsbA* and *DsbC* help optimise disulphide bond formation [34]. The selection of Fabs during panning was greatly improved with much higher levels of Fab detected on phage through each round of panning compared to when the co-plasmid was not present. A study by Levy et al. [36] also used co-expression of the PPIase *FkpA*, but it was retained in the cytoplasm or directed to the periplasm. Functional Fab levels were improved to a greater extent when the *FkpA* was expressed in the cytoplasm. During panning, the system improved the diversity of antigen specific Fabs from 10–16% to 43–48%. The authors speculate that *FkpA* isomerises proline residues in kappa light chains and when subsequently exported to the periplasm this improved folding and Fab assembly. The strategy improved periplasmic soluble Fab levels from 0.4–2.5 mg/L to 3.5–14.2 mg/L.

A different approach displayed single chain Fabs (scFab) where the C-terminal of the light chain was linked via an extended 60 amino acid linker to the N-terminus of the heavy chain [25]. This extended linker allowed retention of the interchain disulphide bond (shorter linkers necessitated the removal of this bond) and effective scFab display. The authors speculated that this approach of tethering of the Fab chains may increase the folding rate. The study also looked at optimising the secretion of the Fabs by routing through the signal recognition particle (SRP) pathway for co-translational secretion and comparing this to the more usually employed secretion of the unfolded post translation scFab proteins. The former used the signal sequence from *DsbA* and the latter the *pelB* signal sequence. The hypothesis was that co-translational secretion would prevent any scFab folding in the cytoplasm that may reduce efficient folding/secretion. In addition, the study also selected optimised mutants of the signal peptides and found 3 mutants of *pelB* and 2 of the *DsbA* signal sequence with improved display levels. Overall, using an optimised *DsbA* signal sequence resulted in a threefold improvement in display to 0.5 scFabs/phage particle. In terms of scFab expression levels, soluble scFab was improved from 1–3 mg/L to 3–4 mg/L when switching to the co-translational secretion system.

**Fig. 2:**
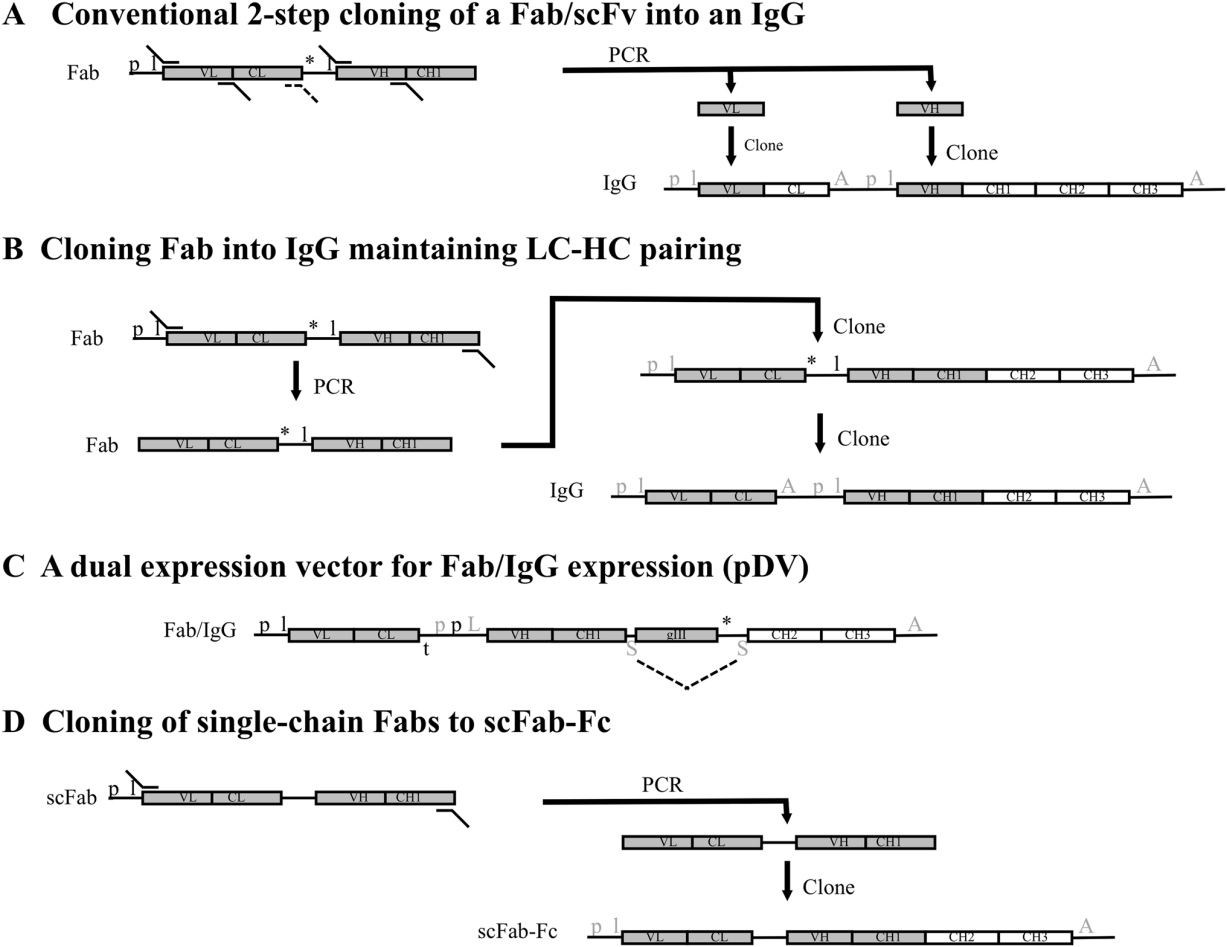
A summary of key elements within different strategies to produce full-length IgG or Fc-fusion constructs from Fab phage display vectors. Conventional cloning of Fab usually includes the amplification of the VH and VL domains and cloning into appropriate sites within an ‘IgG cassette’ that contains the CL and CH1, CH2 and CH3 domains within a mammalian expression vector (a). For Fabs, an analogous 2 step cloning method can be used to clone the entire light chain along with the VH domain (A, dashed primer would be used). The phagemid (left hand side) will contain *E. coli* promoter (p) and leader (l) sequences for the expression of the Fab. The antibody fragment gene is expressed as a fusion with a downstream gIII phage coat protein gene. Once the VL (or light chain) and VH domains are cloned into the IgG expression plasmid, the light and heavy chains are expressed separately with their own eukaryotic promoter (p) and leader (l) sequences and poly(A) tail (A) [mammalian regulatory elements are indicated in grey font]. Fabs have also been cloned from a phagemid vector in two steps into a mammalian expression vector to retain the pairing of the light chain and VH-CH1 domains, allowing batch cloning of polyclonal phagemid [21] (b). In the first step the Fab is amplified and cloned into the vector and then in a second step the mammalian regulatory elements are introduced between the light chain and heavy chain genes. With a Fab library that only contain heavy chain diversity, this can be expressed within Fab or full-length IgG formats from the same vector (c). The pDV system [24] expresses the Fab or IgG when in *E. coli* or mammalian cells, respectively, without any requirement for sub-cloning steps. The construct uses a bacterial p and l for the light chain and has a bacterial transcription terminator (t) after the light chain. The sequence upstream of the VH-CH1 genes contains bacterial and eukaryotic promoters along with a leader sequence that functions in either host cell (L). Downstream of the gIII gene is a stop codon (*) followed by a splice domain (S). A second S is present between the CH1 and gIII domains. In bacteria, Fab-gIII is expressed and in mammalian cells, intron splicing (dashed lines) removes the gIII region and the heavy chain is expressed and paired with an invariant light chain derived from a different vector. Alternatively, single chain Fabs expressed as fusions with pIII can be sub-cloned in a single step into a mammalian expression vector containing the Fc domain to make scFab-Fc [25] (d)

It is often desired to convert Fabs into full-length IgGs as the final antibody reagent. This is often not a straight forward procedure and usually takes 2 steps, each of which sequentially clones the VH and VL (or whole light chain) domains into an expression vector. Either using a single expression vector (Fig. 2a) or more usually by cloning the light and heavy chain domains into distinct vectors [37]. As these conventional reformatting strategies clone the heavy and light chain variable regions independently they are unsuitable for batch cloning, where heavy and light chains would need to retain their pairing. Screening recombinant antibodies from phage display in an IgG format is therefore labour intensive and presents a bottleneck in discovering functional IgGs [37]. Jostock et al. [21] reported vectors that could express Fabs as human IgG1 or IgG4 or mouse IgG2a antibodies. These also allowed Fab regions to be switched between vectors. The cloning strategy retained the pairing of light and heavy chain regions and so could be applied to the batch cloning of Fabs from phagemid into the IgG expression vectors. The method readily recovered ~ 90% of the Fabs, allowing screening to be done with divalent IgGs produced in mammalian cell culture (see Fig. 2b for cloning details). Transient expression in HEK293T cells produced 5–18 mg/L IgG. An alternative approach has been described that uses a vector (pDV) that is compatible with both bacterial expression of Fab and mammalian cell expression of full-length heavy chain without sub-cloning [26] (see Fig. 2c for cloning details). Fab diversity was only within the heavy chain in the pDV library. The system was based on a signal sequence from murine binding immunoglobulin protein (mBIP) that can support secretion of heavy chain sequences in both cell types. A stop codon after the pIII protein prevents the expression of the Fc region within bacteria. The pIII gene and stop codon were sited within an intron and were removed by RNA splicing in mammalian cells allowing expression of full-length heavy chain. Within the mammalian cells an invariable light chain was expressed from a separate vector to allow IgG formation. A library of 10^9^ diversity was produced and panning allowed selection of specific Fabs. Cognate IgGs were transiently expressed and several had affinities in the low nM range. The levels of IgG produced were 2–6 mg/L which is lower than with other expression vector systems but was sufficient for screening antibodies. Alternatively, the scFab system described by Koerber et al. [25] allowed batch cloning in a single step to an scFab-Fc format (scIgG; see Fig. 2d for details), made up of IL2 signal peptide-scFab-rabbit Fc. Transient expression in HEK293T cells yielded 5 mg/L scIgG.

## Single Chain Variable Fragments (scFv)

ScFvs offer high sensitivity, specificity and ease of expression in different systems. Unlike Fabs, scFvs are expressed from a single gene and the variable regions are tethered by a flexible linker, improving both expression and refolding/assembly [38]. However, they can display lower affinity than the corresponding Fab and can have lower long-term stability [39]. The production and utility of scFv-phage libraries and the screening and application of the binders have the same issues as outlined for Fabs. High diversity single-pot libraries are required to produce high affinity reagents against any target [40] and numerous libraries containing greater than 10 billion clones have been reported [41–43]. Consistent, high stability and high expression levels in both bacteria and mammalian systems for all library clones would be a valuable trait to allow the isolation and development of the best binders. As with Fabs, scFvs have relatively high tumour penetration compared to IgG and a rapid in vivo clearance, making them useful tools for in vivo imaging [44–46]. However, many applications require divalency and Fc region availability, and so the batch reformatting of scFvs into IgGs or scFv-Fc formats for screening would be of value [40].

For scFvs it has been reported that the contribution of the distinct domains to the antigen binding site is in the order CDRH3 > CDRH2 > CDRL3 > CDRH1 > CDRL1 > CDRL2 > Framework regions [40]. Naïve libraries are traditionally produced by sequential amplification and cloning of the VL and VH regions into a phagemid vector separated by a (Gly4-Ser)3 linker [47]. They usually then contain c-myc and polyHis tags between the C-terminus of the scFv and the N-terminus of the pIII coat protein. Kugler et al. [43] demonstrated that the order of the tags can influence soluble scFv expression levels in bacteria. They reported yields in the low mg/L range which is similar to those produced after sub-cloning of scFvs into a pET expression vector [48]. As CDRH3 has the highest natural diversity and contributes the most to the antigen binding site [40], synthetic and semi-synthetic libraries invariably target this region for mutagenesis, sometimes along with other CDRs. For example, the Tomlinson I and J library mutated CDR2 and CDR3 regions of both VL and VH [13], the HuCal scFv library mutated CDR3s and the HuCal GOLD library introduced diversity into all CDRs [14–16].

ScFv libraries are usually produced using multiple PCR reactions to amplify diversity and introduce the linker region and then assembly of scFv chains via overlap extension PCR. However, this can result in mis-annealing, particularly in the linker repeat region, and produce non-functional fragments. Nishi et al. [49] used this method to produce a synthetic library with randomised CDR3s which had a diversity of ~ 10^8^ but 8 out of the 13 clones sequenced had errors. Libraries of diversity ~ 10^11^ made by an analogous method contained 66–85% clones with ORFs and 58–85% expressed soluble protein [40]. An alternative method introduced diversity through PCR and cloning of the VH and VL libraries separately into a pUC vector along with partial linker sequences containing a unique restriction site. This is then followed by sub-cloning of the libraries into a phagemid vector to form scFv cassettes without further PCR amplification. This strategy produced much higher levels of functional scFvs, with no errors found in 83 sequenced clones [49].

Weber et al. [50] used single VH and single VL kappa and VL lambda genes as the scaffolds for a synthetic scFv library where they introduced mutations into CDR3s with partially degenerate primers. They also chose asparagine at residue 52 of VH as this mutation is often present due to somatic hypermutation and during affinity maturation. The VH selected as scaffold had high thermal stability and also bound protein A when part of an scFv. The resulting PHILODiamond library had ~ 10^10^ diversity with 90% of clones having a functional scFv. They speculated that as only the CDR3s were mutated, affinity maturation of clones could be carried out by introducing diversity into the CDR1 and 2 loops.

Codon usage for mutagenesis in synthetic or semi-synthetic libraries is often NNN or NNK but can also be more tailored such as the use of NWG, NWC and NSG codons (where N = A/T/G/C, K = G/T, W = A/T, S = G/C) to avoid the introduction of cysteines and stop codons (other than amber) into CDRs [51]. Säll et al. [52] introduced an even more specific mutagenesis strategy. They used trimer phosphoramidite codons to predetermine the diversity introduced into and adjacent to CDRHs and CDRL3. The strategy introduced up to 13 amino acids into any one position and was based on the diversity of commonly found residues in natural repertoires, with particular bias for tyrosine, glycine and serine residues. The strategy also varied the length of the CDR3s. The library used single human scaffold VH and VLs (kappa and lambda) with codon optimisation for expression in bacteria and mammalian cells. Two libraries were produced, HelL-11 and then HelL-13 with diversities of ~ 10^10^. The former library had stop codons in both CDR3s of the scaffold scFv and the resulting library displayed just 47% functional scFvs, it was also found that tryptophan was over-represented within the diversity regions relative to the concomitant codon usage in the oligonucleotide synthesis. The HelL-13 library was adjusted to remove tryptophan bias and also used a scaffold scFv with a stop codon only in CDRH3. This resulted in 60% of clones having a functional scFv and it was found that functional scFvs selected against various targets paired the scaffold CDRL3 with numerous CDRH3s.

A semi-synthetic human scFv library (ALTHEA Gold) has been produced that selected for highly diverse thermostable scaffolds before introducing a natural CDRH3 diversity [53]. Firstly, two kappa VL domains and a single VH domain had diversity introduced using trinucleotide phosphoramidites. These mutations coded for amino acids found in natural repertoires at 10 positions within the VH (within CDRH1 and CDRH3) and at 10 or 12 positions within the two VLs (within all 3 CDRs). Further diversity was then added by cloning 90 neutral CDRH3 containing fragments, resulting in a library of ~ 1 × 10^9^ diversity. The selection for high-stability scFv-phage clones was then carried out by heating the library for 10 min at 70 °C and purifying stable, native scFvs on protein A (that binds the particular VH used). This thermostable library of scaffolds was then further diversified by the introduction of natural CDRH3 fragments from 200 donors. The resulting library had a diversity of ~ 10^10^. Binders were selected from the library and once converted to IgG they displayed affinities in the very low nM to pM range. These IgGs were produced at 50–90 mg/L in HEK293 cells.

An alternative approach to scFv library construction has been recently described by Zhao et al. that used the modular cloning of different CDR regions [54]. Antibody sequence databases were mined for heavy and light chain CDR2 and CDR3 sequences contained within a particular scFv framework. The rationale was that such modular domains are known to be functional and the approach would avoid non-functional clones present in synthetic/semi-synthetic libraries. Non-functional clones could arise due to incompatibility between CDR mutations and the framework scaffold(s) and/or incompatibility between amino acid combinations within highly mutated CDRs. Based on 2000 scFv sequences, shuffling of the 4 CDR regions produced a library of ~ 10^10^ diversity. This so-called predefined CDR sequences (PDC) library was estimated to produce 20-fold more unique functional scFvs per target compared to libraries produced with degenerate oligonucleotide methods.

As with Fabs, in silico programmes that can predict antibody solubility can also be applied to phage-scFv panning outputs. The aim being to select high affinity binders that also have high solubility without the need for extensive in vitro testing. Sormanni et al. compared the CamSol programme to in vitro solubility tests using nine IgGs derived from scFvs isolated by phage display [55]. Hotspots linked to aggregation were identified within the scFv domain and solubility could be accurately predicted. Such in silico stability prediction tools have now been validated with Fabs and scFv domains to improve lead antibody identification.

**Fig. 3:**
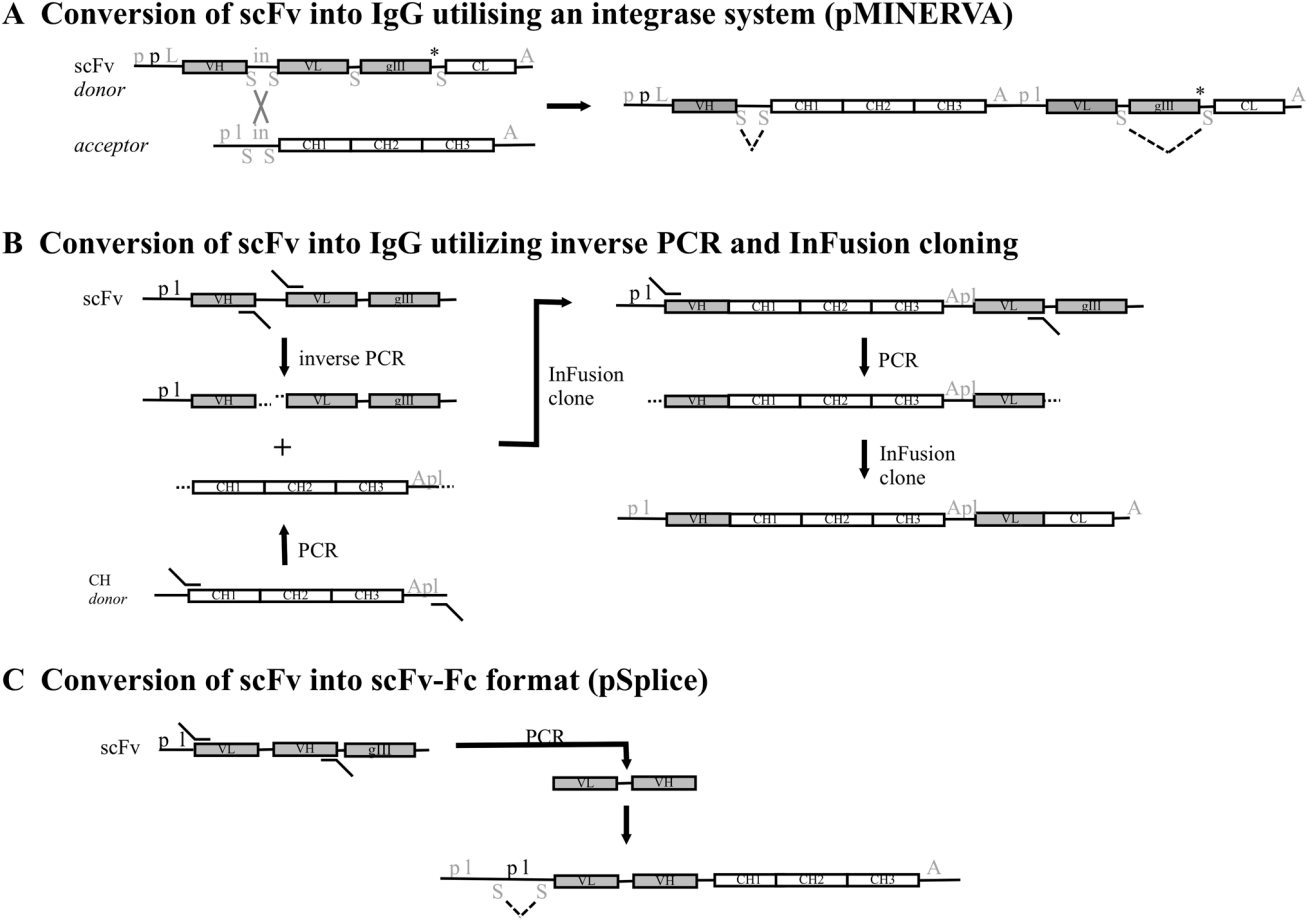
A summary of key elements within different strategies to produce full-length IgG or Fc-fusion constructs from scFv-phage display vectors. A conventional 2-step cloning strategy for scFvs is analogous to that for Fabs shown in Fig. 2a. Alternatively, the pMINERVA system [58] utilises both bacterial integrases and also intron splicing in mammalian cells (a). A phagemid ‘donor’ vector contains mammalian (grey font) and bacterial (black font) promoters and a leader sequence compatible with both cell types (symbols are the same as in Fig. 2), in addition the scFv has a linker that is also the substrate for phiC31 integrase (in). The construct also contains splice sites either side of the integrase substrate region and either side of gIII. The gIII has an ochre stop at its 5’ end followed by the CL domain. The construct can be transferred to an *E. coli* strain that expresses the integrase and where a second acceptor plasmid is present that contains the CH domain (CH1-CH2-CH3) and regulatory elements (p and l) for mammalian expression of the light chain. Integrase mediated recombination results in an IgG expression vector where, upon transfer to mammalian cells, intron splicing (dashed lines) results in the removal of the gIII region and expression of light chain and heavy chain. Alternatively, InFusion technology, allowing the cloning of overlapping 15 bp sequences, has been used in a multi-step cloning strategy to produce IgG from scFv [59] (b). The phagemid was amplified by inverse PCR to produce a linear version of the whole vector. This step removed the scFv linker and added an overlapping sequence (indicated by …). This same overlapping sequence was added in a separate PCR reaction to a DNA fragment containing CH domains and mammalian promoter and leader sequences (amplified from a ‘donor’ vector). InFusion cloning produced the heavy chain and VL region, the latter with mammalian regulatory elements, that are then amplified by PCR and subsequently cloned using InFusion into a mammalian expression vector containing the regulatory elements for the heavy chain as well as the CL domain. ScFv from phagemid can potentially be batch cloned into a vector (pSplice) that can express scFv-Fc in both bacterial and mammalian cells [56] (c). The scFv could be amplified by PCR and cloned into the pSplice vector that contains a mammalian promoter and leader sequence in addition to *E. coli* promoter and leader sequences, all upstream of the scFv-Fc coding region. The mammalian leader sequence is incomplete with the 3′ region contained after the prokaryotic p l sequences that are flanked by splice domains. The scFv-Fc is expressed from the cassette in *E. coli* and in mammalian cells the intron containing the bacterial regulation elements is excised (dashed lines) and the mammalian leader sequence spliced together allowing expression of the scFv-Fc

As with Fabs, the reformatting of scFvs into IgGs is important for various applications including the development of therapeutic antibodies. Standard methods involve the sequential cloning of VL and VH genes into appropriate IgG expression cassette vector(s), analogous to that for Fabs (Fig. 2a). Alternative methods that allow the batch cloning of scFv sub-libraries after panning selection would be far less labour intensive than cloning individual clones. Such methods would also allow the screening of IgGs for function and therefore facilitate the selection of antibodies with the best properties for the end application. This latter trait has been termed ‘screening in product format’ (SiPF) by Xiao et al. [56]. As with Fab cloning, the challenge is maintaining the VL-VH pairing during the batch reformatting. Liu and co-workers retained VL-VH combinations when reformatting an scFv sub-library into an IgG format by using overlap extension emulsion PCR [57]. The method produced 1 × 10^4^ IgGs from a panning sub-library and the diversity and relative abundance of clones correlated between the original scFv library and the IgG resultant library. Batonick et al. [58] developed a system to reformat scFv into IgG that takes advantage of both bacterial integrases and also intron splicing in mammalian cells. Their system, termed pMINERVA, uses a phagemid ‘donor’ that contains mammalian and bacterial promoters and an scFv with a linker between the VH and VL genes that is also the substrate for phiC31 integrase. The construct also contains splice sites either side of the *gIII* gene. The *gIII* gene has an ochre stop at its 3′ end followed by a splice site and the CL domain (Fig. 3a). The vector containing the scFv of interest is transduced into an *E. coli* strain that expresses the integrase and contains an ‘acceptor’ plasmid. This plasmid contains the CH domain as well as regulatory elements for mammalian expression (of the light chain). The plasmids undergo specific recombination resulting in an IgG expression vector where, in mammalian cells, the gIII region is removed by intron splicing resulting in expression of light chain and heavy chain (Fig. 3a). Efficacy was demonstrated for a single scFv and the method may allow batch reformatting of scFvs of the original library produced in the ‘donor’ phagemid. An alternative system also allowing batch reformatting uses inverse PCR to amplify VH and VL within a whole phagemid, effectively producing linear phagemid and removing the linker sequence [59]. This also introduces complementary overhangs. The latter are also used in the separate amplification of a donor fragment containing a hinge-CH region, translation stop and polyA sites and the control elements for light chain expression (promoter and leader sequence). InFusion [60] technology is then used to fuse this donor fragment into the phagemid. This IgG cassette is then PCR amplified and cloned into a mammalian expression vector containing the promoter and signal sequence for the heavy chain, the CL domain (either kappa or lambda) and a polyA site (Fig. 3b). When using emulsion PCR to limit recombination between clones, the accuracy of this batch reformatting was 90% in terms of VH-VL pairing. The study suggested that reformatting retained the relative abundance of clones in an scFv pool and when reformatting 25 scFvs, 84% were successfully obtained as IgGs (the missing clones were present just once in the scFv pool when 129 clones were sequenced). Furthermore, when assessing the expression levels of the IgGs, in 293F cells, clones produced 0.4–40 mg/L IgG. When using Expi293F cells, expression levels were between 1 and > 200 mg/L for the 1500 clones assessed. The system is compatible with any pre-existing scFv library and results in high fidelity batch reformatting and consistent, high-level IgG production.

An alternative to converting scFvs into IgGs is to produce scFv-Fc fusions by cloning the scFv into a mammalian expression vector containing the Fc domain in a single step. This format has been shown to produce 10–20 mg/L in 239T cells, which can be optimised to up to 600 mg/L [61]. Similarly, Xiao et al. [56] developed a vector that allows both bacterial and mammalian expression of scFv-Fc fusions. The system, pSplice, is based on a vector that contains mammalian expression control elements followed by an intron coding for a bacterial promoter and signal peptide and then the scFv-Fc gene. In mammalian cells, the intron is spliced out to produce a functional operon for scFv-Fc (Fig. 3c). This vector system is designed to allow the rapid screening of scFv-Fc using bacterial expression before the transfer of selected clones to mammalian cells. Both systems have the potential to batch clone scFv pools after panning to allow the screening of bivalent antibodies.

Another recent advance in scFv-phage display technology is the production of functional, renewable polyclonal scFv reagents. Polyclonal antibodies produced in vivo are not renewable and can often contain as little as 0.5–5% target-specific antibodies [62]. The strategy to combine the use of phage and yeast display to screen for binders was described by Ferrara et al. [63]. Two rounds of phage selection against a target were followed by PCR amplification of the scFv sub-library (representing ~ 10^5^–10^6^ clones) and batch cloning into a yeast surface display vector. Two rounds of fluorescence activated cell sorting resulted in ~ 63% of scFv clones binding the target and all scFvs had low background binding. This polyclonal scFv reagent was highly specific and could be regenerated after dilution of over a 100 million-fold without loss of function or relative abundance of the clones. The affinities of the reagents were ~ 50–500 nM which reflected the concentration of antigen that was used in the panning. Such a strategy may have advantages over generating monoclonal ligands when targeting complex antigens presenting multiple epitopes.

## Human Single Domain Antibodies

Whilst far less utilised than scFv or Fab phage display libraries, several single domain human antibody libraries derived from either VH or VL regions have been described and characterised [64, 65]. These are relatively easy to produce as synthetic or semi-synthetic libraries. However, the disadvantages of these antibody formats are the unreliable isolation of target-specific clones by phage display, relatively low stability, low solubility and often relatively poor affinities [64, 65]. Henry et al. [66] attempted to address these limitations by extensively screening human VH and VL scaffolds to isolate those with high stability, solubility, yield and suitability for carrying CDR mutations. They then selected three of the most suitable scaffolds and carried out targeted trinucleotide mutagenesis of CDRs producing libraries of ~ 10^10^ diversity. High affinity ligands (low nM) were isolated but the general limitations of human single domain antibodies were not resolved. These issues may be due to human VH and VL domains usually being paired in nature resulting in the burying of hydrophobic surfaces that are surface exposed in the equivalent single domain structures.

## Camelid V_H_H Nanobodies

V_H_Hs are derived from heavy chain antibodies (HCAbs) as first described in 1993 [67] and consist of the equivalent of a conventional IgG VH domain (Fig. 1). HCAbs are naturally found in camels, alpacas and llamas where they make up a significant proportion of the IgG population. For example, in alpacas IgGs occur in a ratio of approximately 5:3:2 for IgG1 (conventional IgG structure):IgG2 (V_H_H with a short hinge length):IgG3 (V_H_H with a long hinge length), and so HCAbs can make up approximately 50% of circulating IgG [68].

Similarities between V_H_H and the VH domain of a typical IgG are the presence of a disulphide bond between framework regions (FRs) that stabilises the structure, as well as it being composed of 3 CDR regions held by scaffold FRs with beta-sheet structure. Differences between the VH domain and V_H_H include the latter often having an addition disulphide bond between CDR1 and CDR3 that reduces the flexibility of the paratope, the CDRs 1 and 3 are generally longer in V_H_H and its FR2 region is more hydrophilic. This latter property prevents the FR2 region interacting with a light chain and also results in higher solubility compared to the VH domain [69–71]. V_H_Hs usually have relatively high stability and solubility and can very effectively refold after heat denaturation. Their high stability means they often have a high tolerance to organic solvents [72] and temperature making them very adaptable reagents in assays. V_H_Hs also have some unusual binding characteristics in that the reduced size of their paratope (made up of 3 rather than 6 CDR regions) with an often extended CDR3 leads to effective binding within ‘pockets’ such as enzyme active sites. However, V_H_H binders have been identified to a wide range of antigens including more ‘flat’ structures [20, 68]. It has been suggested that a relatively small paratope may be a disadvantage when binding haptens, and whilst in vivo camelid antibodies against such antigens tend to be dominated by IgG1, there are numerous examples where phage displayed V_H_Hs have been isolated to haptens [20], indicating they have a broad range of potential epitopes. When considering V_H_Hs as in vivo reagents, their small size may allow superior tissue/tumour penetration compared to larger antibody formats, their lack of an Fc region means they provoke less immune effector responses and they have a relatively rapid distribution and subsequent clearance. These traits indicate that they are good candidates as ligands for producing high contrast images [72]. Indeed, single domain antibodies have already been developed into various molecular imaging tracers [73–77]. V_H_Hs with potential therapeutic applications have also recently been described [78–80].

The isolation of V_H_Hs often involves the immunisation of camelids and the subsequent production of an immune phage display library. Obvious limitations of this approach are that each library is only valid for a single (or limited number) of antigens, the cost of keeping the animals is high and toxic antigens cannot be used. However, there are now in vitro alternatives to this approach. One strategy that has been recently demonstrated is to stimulate camelid B cells in vitro with antigen in the presence of IL2 and IL4 and then clone a small V_H_H display library of ~ 10^6^ repertoire [81]. Alternatively, single-pot V_H_H phage display libraries have been produced and range in diversity from ~ 10^7^ to ~ 10^9^ [18, 69, 82–85]. These can be produced from natural diversity or by engineering diversity into the CDR regions of a scaffold V_H_H. For example, Sabir and co-workers amplified and cloned the natural diversity of V_H_Hs for both IgG2 and IgG3 producing a library with 10^7^ diversity [69]. For synthetic libraries, Yan et al. [18] used the cAbBCII10 V_H_H template as an effective scaffold for grafting in CDR diversity, this scaffold has high stability and expression levels and lacks the CDR1-CDR3 disulphide bond [86]. They introduced a 16-amino acid random insert into the CDR3, using the NNK codon, to produce a 10^9^ library. Cloning used an overlapping PCR method to amplify the V_H_H gene with the overlap in the FR3 domain. Interestingly, V_H_Hs isolated from this library against distinct targets had very different expression levels in bacteria indicating the significant contribution of the CDR3 region to antibody yields.

The expression levels of V_H_Hs is relatively high in both bacterial and eukaryotic expression systems and this is often accompanied by high thermal stability. For example, bacterial expression often produces > 5 mg/L of V_H_H [18, 71, 86]. Studies have looked at further improving thermal stability [87, 88] by the introduction of an extra disulphide bond between FR2 and FR3. This approach usually increased thermal stability but often resulted in lower expression levels and in one case also reduced refolding after heat denaturation [71]. An additional strategy was to then introduce negatively charged amino acid substitutions into FRs to lower the isoelectric point of the V_H_H. This was shown to improve expression and refolding properties for some antibodies [71], however, it did not increase yields for all V_H_Hs with the extra disulphide bond. Overall, this strategy presents an additional or alternative approach for the stabilisation of V_H_Hs.

In summary, V_H_Hs have greater stability than other antibody formats and are expressed at relatively high levels in bacteria. Their smaller size and smaller paratope does not seem to constrain the binding properties of V_H_Hs isolated in vitro. V_H_Hs may also offer advantages when targeting ‘buried’ epitopes such as the active sites of enzymes. Compared to other antibody formats, V_H_Hs may also offer improved tumour penetration for in vivo applications. Improving V_H_H in vivo properties such as effector function and increased serum half-life may be achieved by fusion to an Fc domain. This has been achieved for individual V_H_Hs [89] and should be feasible for batch reformatting of V_H_Hs from phage display experiments in analogous procedures to those for scFv-Fc and scFab-Fc formats.

## Shark V_NAR_ Nanobodies

There are relatively few examples of V_NAR_ phage display libraries compared to scFv, Fab and V_H_H formats. The V_NAR_ structure is analogous to V_H_H in that it has a relatively long CDR3 region of up to 40 amino acids, averaging ~ 18 residues [19, 90] and this region represent most of the diversity. They also have disulphide bonds that stabilise their structure (including a canonical bond between FR1 and FR3), and are relatively small at ~ 12 kDa. Similar to V_H_Hs, V_NAR_s are highly stable. However, V_NAR_s do not have a CDR2 region, instead they have 2 mutation-prone regions between CDR1 and CDR3 termed HV2 and HV4 [19, 91]. The V_NAR_s are classified as Type 1 to Type 4 depending on the number and location of non-canonical disulphide bonds [18, 91]. Type 1 contains 2 cysteine residues in CDR3 that form bonds with residues in FR1 and FR4, type 2 have a bond between CDR3 and CDR1, type 3 are similar to type 2 but with a conserved tryptophan adjacent to the bond location in CDR1 and type 4 have none of the non-canonical bonds that are seen in the other 3 types. Feng et al. also describe further types of V_NAR_s that are yet to be classified [19]. Type 1, 2 and unclassified V_NAR_s with specific binding properties have been isolated from phage display experiments [19, 90, 92–94]. V_NAR_s are from a single gene family and so are very easily cloned, just 3 primers in 2 PCR reactions have been used to clone V_NAR_ diversity [90]. Both immune and single-pot phage libraries have been produced. Immune libraries of ~ 10^6^ to 10^7^ diversity have been reported [90, 94, 95], one of which yielded an antibody with low nM affinity [90]. Up until very recently, single-pot libraries were produced from a range of shark species but with relatively low diversities of ~ 10^7^ [92, 93, 95, 96]. These libraries were either synthetic [92, 97] based on up to 26 scaffold sequences and randomised CDR3, a mixture of synthetic CDR3 diversity and natural diversity [93] or had completely natural diversity [96]. A naïve library with high diversity has recently been produced by Feng et al. [19]. They used a method termed PCR extension assembly and self-ligation (EASeL) to produce a library with diversity of ~ 1 × 10^10^. The method amplified the V_NAR_ gene region and a phagemid vector separately, both with a complementary overhang. Both products were then amplified together in a subsequent extension PCR, followed by self-ligation. The library was used to isolate binders to diverse targets and one binder had an affinity of 10 nM. Across all studies, the V_NAR_s could be produced in *E. coli* but with varying levels of expression, between ~ 0.2 and 15 mg/L [19, 93]. Zadeh et al. has also produced a synthetic V_NAR_ library based on the anti-lysozyme HEL-5A7 V_NAR_ clone, introducing diversity into a 12 to 23 amino acid CDR3 [98].

As with V_H_Hs, batch reformatting of V_NAR_s into IgG-like structures to improve in vivo properties has not been reported. However, individual V_NAR_s have been converted into V_NAR_-Fc fusions [99]. Batch reformatting of this type of antibody should therefore be feasible using analogous procedures to those for scFv-Fc and scFab-Fc formats.

## Conclusions and Future Perspectives

This review has considered the phage display of Fabs, scFvs and nanobodies. With potential application to all recombinant antibody library types are the recent improvement in library design and display efficiency. The diversity within the CDR regions can now be designed with considerable accuracy by using trimer phosphoramidine codons to mimic natural amino acid usage [52]. Furthermore, improvements have been made in reducing cloning errors due to the presence of linker repeat regions [49]. Both advances will facilitate the optimal cloning of diversity into libraries in the future. The co-expression of chaperones and isomerases [34, 36] as well as the use of the co-translation secretion pathway with optimised signal sequences [25] can significantly improve antibody phage display efficiency, thereby improving the efficiency of isolating binders. These same strategies also improve soluble antibody expression levels. Furthermore, the identification and use of highly stable Fabs and scFvs as scaffold for the introduction of synthetic diversity have improved expression and stability of the resulting antibody fragments [31, 32, 50, 66]. The combined use of these strategies to yield higher expression levels and increased stability has the potential to produce more efficient phage display of antibodies and therefore improve panning efficacy. They should also produce more useful reagents.

Methods suitable for the batch reformatting of both Fabs and scFvs into IgGs for high-level expression in mammalian cell culture are now also described [26, 27, 58, 59]. These will facilitate the efficient cloning of sub-libraries of antibody fragments from panning experiments to allow them to be screened as IgGs. This has previously been a bottleneck in developing therapeutics from phage display libraries, as the monovalent recombinant antibody fragments often have very different properties to their concomitant divalent IgGs.

Nanobodies from both camelids and sharks are readily amenable to the production of simple, high diversity phage display libraries [18, 19]. They provide small antibodies with very high stability, therefore potentially offering unique properties for diagnostic or even therapeutic applications. In addition, the production of renewable polyclonal scFv from phage display experiments has now been demonstrated and may also provide a new type of diagnostic reagent [63].

Overall, improved methods for the accurate cloning of libraries, more efficient display of antibodies on phage, improved expression levels for soluble antibodies and the ability to screen in multiple antibody formats has the potential to significantly improve the application of phage display to generate an ever-wider range of diagnostic and therapeutic antibodies.
